# Laboratory Aspects and Diagnostic Challenges in a Case of Hypereosinophilic Syndrome

**DOI:** 10.7759/cureus.101679

**Published:** 2026-01-16

**Authors:** Vanessa Rossi, Silvia Velocci, Sergio Bernardini, Maria Morello

**Affiliations:** 1 Department of Experimental Medicine, Tor Vergata University, Faculty of Medicine, Rome, ITA

**Keywords:** automated hematology, hypereosinophilia, hypereosinophilic myocarditis, idiopathic hypereosinophilic syndrome (i-hes), pathology and clinical biochemistry

## Abstract

Hypereosinophilia is defined by a persistent increase in eosinophil levels and can lead to multi-organ damage with highly variable symptoms. The most common manifestations involve the skin, lungs, and gastrointestinal tract, while cardiac and neurological complications can be particularly severe and sometimes fatal. Within this context, we describe the case of a 35-year-old woman with hypereosinophilic syndrome (HES), whose symptoms began during pregnancy. Three months after giving birth, she contracted SARS-CoV-2, which resolved without complications. However, shortly thereafter, laboratory tests revealed marked leukocytosis accompanied by a rapid clinical deterioration. At admission, the patient displayed severe hypereosinophilia associated with systemic inflammation, liver injury, myocardial necrosis, and multi-organ involvement affecting the kidneys, brain, heart, and lungs. As her neurological condition worsened, she required admission to the intensive care unit, where she unfortunately died four days later. The aim of this case report is to raise awareness among clinicians and laboratory professionals about the crucial role of the laboratory finding in assessing disease severity and guiding the diagnostic challenges of idiopathic syndrome (I-HES). The exclusion of secondary causes confirmed the idiopathic nature of the condition. Finally, the case highlights the importance of early recognition of unexplained eosinophilia and systemic symptoms, as timely intervention may be life-saving in rapidly progressive presentations.

## Introduction

Eosinophilia refers to an elevated number of eosinophils in peripheral blood, exceeding the upper limit of the normal reference range. Among the spectrum of eosinophilic disorders, hypereosinophilic syndrome (HES) represents a group of rare conditions characterized by persistent and marked eosinophilia associated with organ involvement or dysfunction. The estimated incidence of HES is approximately 0.036 cases per 100,000 individuals per year [1]. Although the median age at diagnosis typically falls between 20 and 50 years, cases with pediatric onset, although uncommon, have also been reported [2,3].

The severity of eosinophilia is commonly stratified into three categories: mild (absolute eosinophil count just above 1.5×10^9^/L), moderate (1.5-5×10^9^/L), and severe (>5×10^9^/L) [4,5].

The differential diagnosis of eosinophilia is broad and encompasses a range of conditions. The most frequent causes of eosinophil counts exceeding 1.5×10^9^/L include allergic and atopic disorders, parasitic and non-parasitic infections, autoimmune and connective tissue diseases, vasculitis, sarcoidosis, neoplasms (such as carcinomas and sarcomas), and drug reactions [6,7].

The laboratory approach to the diagnosis and management of HES includes: blood tests (complete blood count, peripheral blood smear, and serum markers such as tryptase, vitamin B12, and IgE); bone marrow examination (to assess eosinophil maturation and identify genetic abnormalities); imaging techniques (to detect organ involvement); and specialized tests (for genetic mutations, allergies, or infections). Continuous monitoring is essential to track disease progression and response to therapy. These investigations enable an accurate diagnosis, proper classification of the HES subtype, and the development of an appropriate treatment plan [8].

Various classification schemes have been proposed to better define and manage HES. The 2006 clinical phenotype-based classification divides HES into idiopathic (I-HES), myeloproliferative (M-HES), lymphocytic (L-HES), overlap, associated, and familial variants. Later, the World Health Organization (WHO) refined the classification of eosinophilic disorders within the broader context of myeloid neoplasms in 2008, with reaffirmation in 2016 [9,10]. These reports underscore the importance of laboratory and molecular studies to distinguish "myeloid/lymphoid neoplasms with eosinophilia" harboring specific genetic rearrangements, such as platelet-derived growth factor receptor alpha (PDGFRA), platelet-derived growth factor receptor beta (PDGFRB), fibroblast growth factor receptor 1 (FGFR1), or pericentriolar material 1-Janus kinase 2 (PCM1-JAK2), from chronic eosinophilic leukemia, not otherwise specified (CEL-NOS). It also provides a clearer separation between L-HES and I-HES [11].

## Case presentation

A 35-year-old woman with obesity presented in winter 2024 to a peripheral hospital with severe fatigue, persistent diarrhea, and gastrointestinal symptoms, including abdominal discomfort and altered bowel habits. Her medical history was notable for an uneventful full-term pregnancy, during which she developed a pruritic rash and leukocytosis.

Three months postpartum, the patient contracted SARS-CoV-2 infection and was hospitalized for ten days, with favorable clinical resolution. Shortly thereafter, she experienced a rapid clinical deterioration characterized by progressive systemic symptoms, prompting hospital admission.

At admission, laboratory investigations revealed marked leukocytosis. As shown in Table 1, the complete blood count revealed marked eosinophilia, accompanied by normal-high levels of Hb and mean corpuscular volume (MCV) within the reference range. Severe leukocytosis was evident, driven mainly by elevated white blood cells and neutrophils, while lymphocyte counts remained normal. Thrombocytopenia was noted, reflected by reduced platelet levels.

**Table 1:** Laboratory investigations performed at the time of admission Eos - eosinophils; Hb - hemoglobin; MCV - mean corpuscular volume; WBC - white blood cells; Neu - neutrophils; Lym - lymphocytes; PLT - platelets; IgE - immunoglobulin E

Parameters	Values	Reference range
Eos	31.440 cells/μL	0-500 (cells/μL)
Hb	16.6 g/dL	12.00-16.00 (g/dL)
MCV	83.1 fL	80.0-100 (fL)
WBC	105.840 cells/μL	4.30-10.80 (cells/ μL)
Neu	69.520 cells/μL	1.500-7.000 (cells/μL)
Lym	2.720 cells/μL	1.000-2.000 (cells/μL)
PLT	104.000 cells/μL	150-450 (cells/ μL)
Total IgE	103 IU/mL	0.0-87.0 (IU/mL)

Initial treatment with hydroxyurea (400 mg) and boluses of methylprednisolone was initiated, but the patient showed no significant clinical or laboratory response.

**Figure 1:**
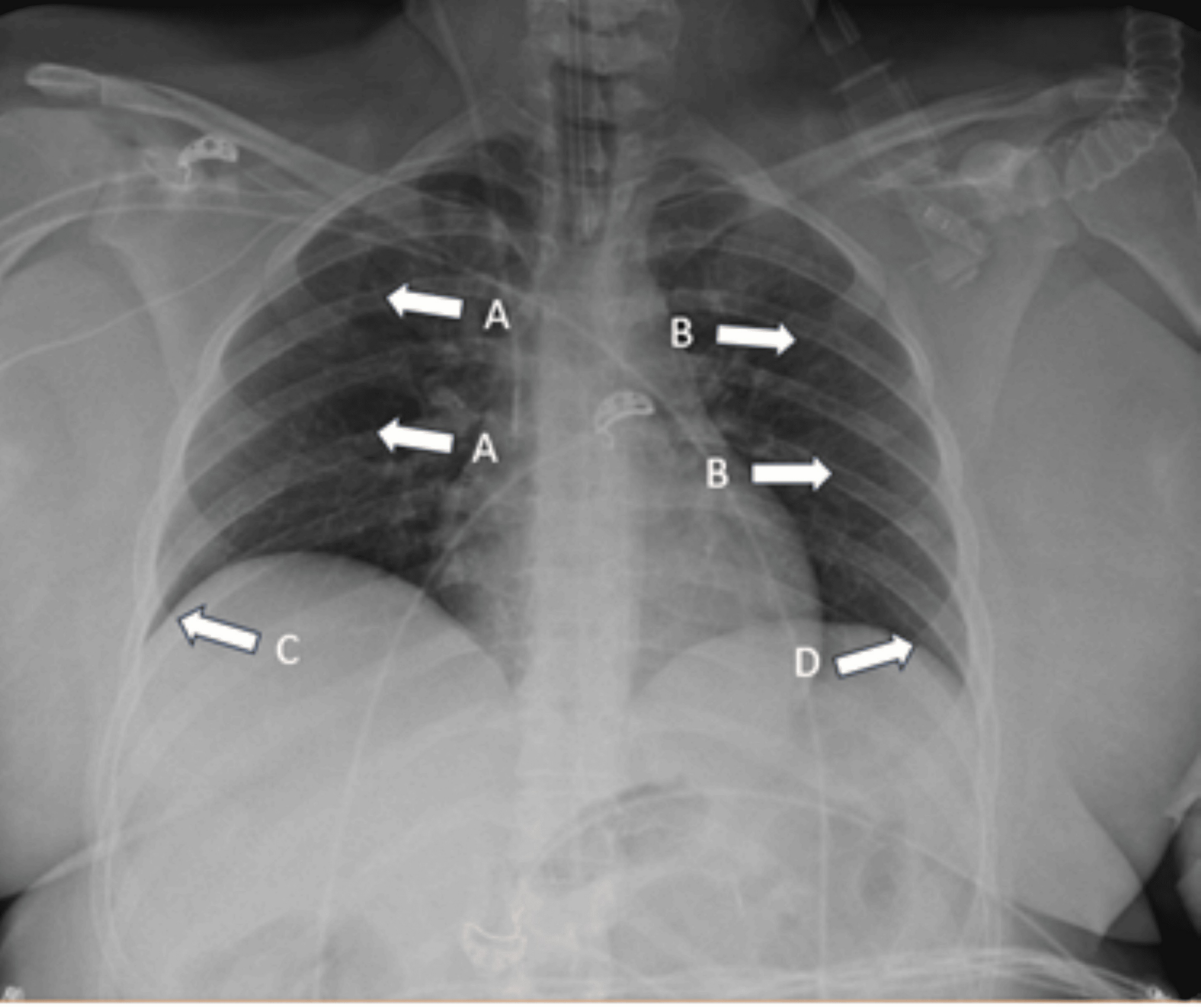
Chest X-ray Examination performed with digital technique in a single AP projection in the supine position. No parenchymal alterations with the characteristics of ongoing inflammation were observed in the bilateral lungs. The lateral costophrenic sinuses were normally expanded. The cardiac image was within normal limits.

Cardiac involvement was identified, with markedly elevated troponin levels (130,000 ng/L), regional wall motion abnormalities, and pericardial effusion, consistent with eosinophilic myocardial involvement. CT performed at the referring hospital demonstrated diffuse lung opacities, upper-lobe consolidations, and hepatosplenomegaly. A subsequent chest X-ray performed at our institution confirmed pulmonary involvement, showing bilateral interstitial thickening with perihilar distribution (Figure 1).

Despite empiric antibiotic therapy, extensive infectious and autoimmune workup remained negative. Bone marrow examination and molecular analyses excluded breakpoint cluster region-abelson tyrosine kinase (BCR-ABL) and factor interacting with PAPOLA and CPSF1-platelet-derived growth factor receptor alpha (FIP1L1-PDGFRA) rearrangements.

Approximately twelve days after admission, the patient developed acute neurological deficits. Brain MRI revealed ischemic lesions suggestive of microangiopathy or vasculitis. High-dose corticosteroid therapy was administered, and follow-up echocardiography confirmed apical akinesia with extensive.

**Figure 2:**
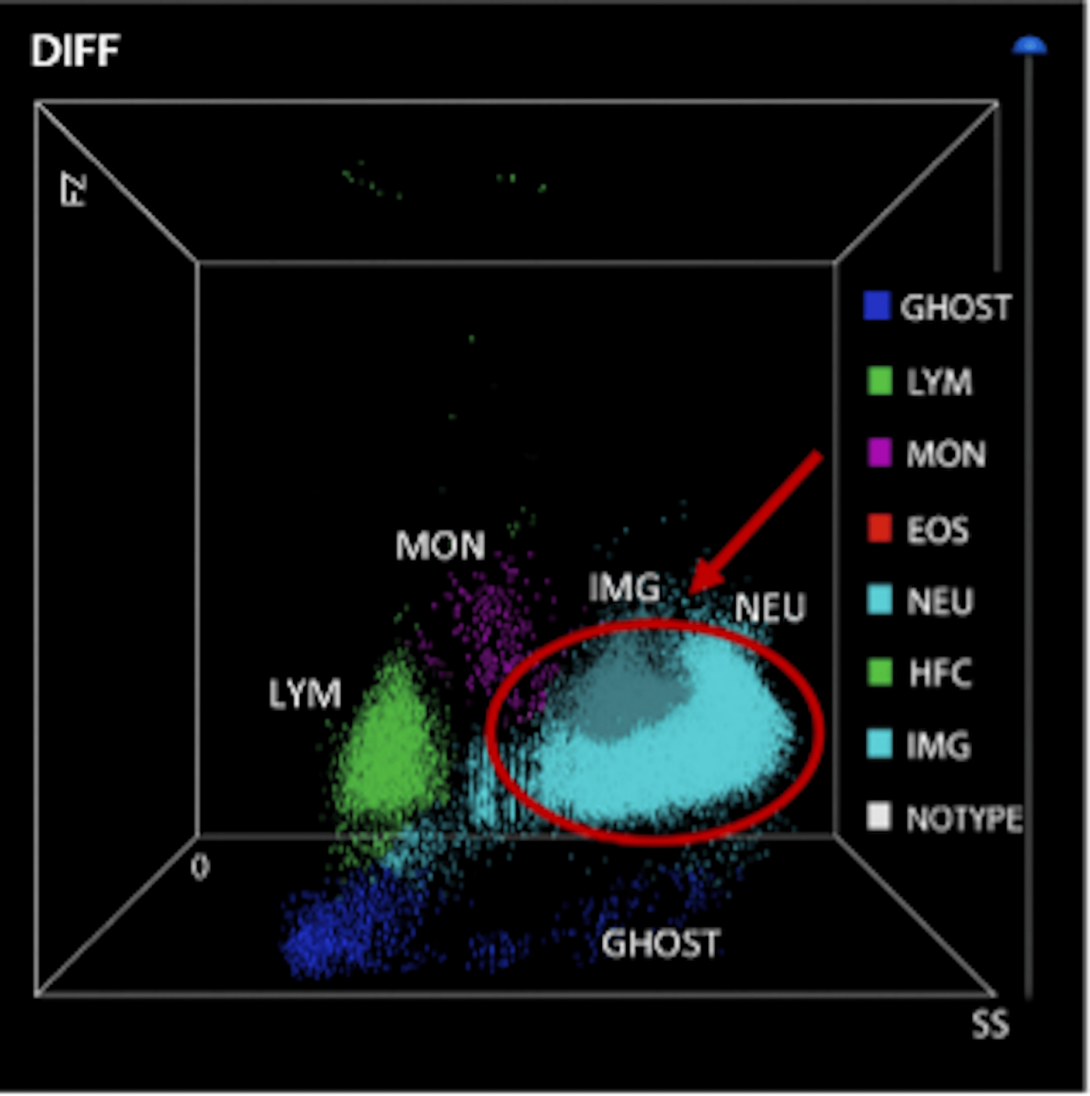
3D scatterplot illustrates the distribution of leukocyte populations obtained through an automated differential count The light-blue cluster highlighted by the red arrow corresponds to neutrophils, positioned in their characteristic region defined by intermediate scatter signals and granularity. Hematological analyses were performed using the Mindray BC-6800 automated hematology analyzer (Mindray Bio-Medical Electronics Co., Ltd., Shenzhen, China). GHOST - ghost events, debris or non-cellular/fragmented events; LYM - lymphocytes; MON - monocytes; EOS - eosinophils; NEU - neutrophils; HFC - high fluorescent cells, typically plasma cells or highly activated cells; IMG - immature granulocytes; NOTYPE - unclassified events

**Figure 3:**
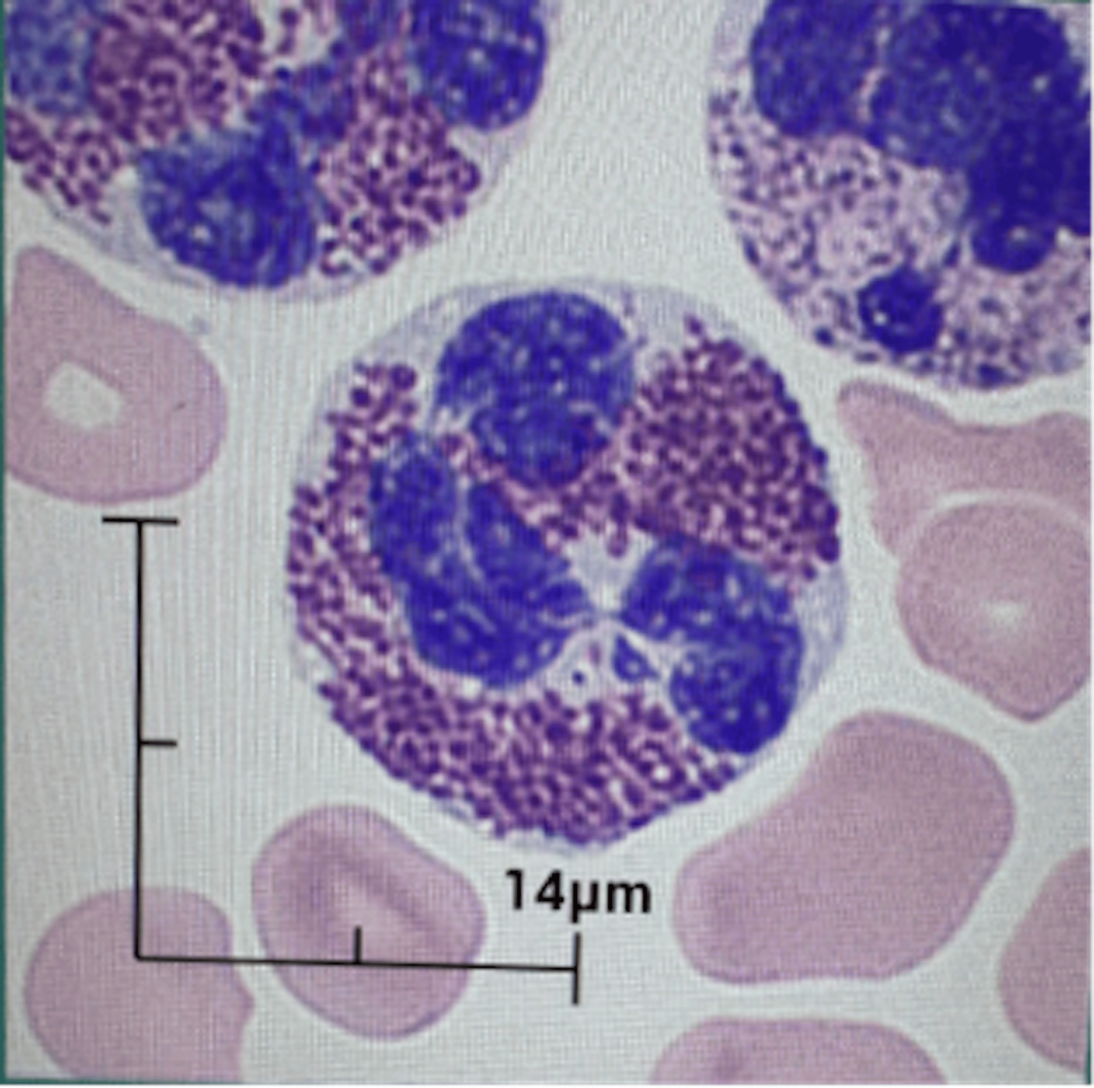
Peripheral blood smear stained with May-Grünwald-Giemsa staining. highlighting eosinophils with hypersegmented nuclei (100X)

After two weeks of hospitalization, laboratory findings demonstrated worsening systemic inflammation, persistent severe eosinophilia, anemia, and thrombocytopenia, with evidence of multi-organ damage. Automated leukocyte differential analysis showed a predominance of cells classified as neutrophils; however, peripheral blood smear confirmed persistent eosinophilia (approximately 90%) without circulating blasts (Figures 2-3).

The patient's clinical condition rapidly deteriorated, requiring endotracheal intubation and transfer to the intensive care unit. She developed multi-organ failure involving the liver, kidneys, and heart. Mechanical circulatory support with a left ventricular assist device (Impella, Abiomed, Inc., Danvers, USA) was required, and therapy was escalated to include imatinib (100 mg/day) and higher-dose hydroxyurea (500 mg/day). Serum protein studies revealed a monoclonal IgG lambda component detected by isoelectric focusing.

Despite aggressive, supportive, and targeted therapy, the patient's condition continued to worsen, leading to a fatal outcome four days after ICU admission.

## Discussion

This case report underscores the important role of laboratory diagnostics in the differential diagnosis of hypereosinophilia. After excluding secondary causes such as infections and malignancies through comprehensive testing, a marked hypereosinophilia (90% of white blood cells) was observed, prompting molecular and cytogenetic analyses to identify the specific form of HES.

In fact, scatter analysis classified a broad cellular gate as "neutrophils", likely due to the atypical and complex morphology of the patient's eosinophils (Figure 2). The previously scatter plot classification was corrected after the study of peripheral blood smear that revealed the abundance of mature eosinophils with dysplastic features, including irregular hypersegmentated nuclei, cytoplasmic vacuoles, and coarse, variably staining granules (Figure 3). Although not definitive, eosinophil dysplasia may indicate either reactive or clonal eosinophilic proliferation and should prompt further molecular evaluation. Given that these dysplastic features can be observed in both reactive and neoplastic eosinophilic conditions, it is important to recommend comprehensive investigations to assess the clonality or the presence of an underlying myeloid neoplasm [12]. 

Eosinophilic neoplasms associated with molecular alterations are rare and heterogeneous entities characterized by specific genetic aberrations involving tyrosine kinase receptor genes such as PDGFRA (4q12), PDGFRB (5q31-q33), and FGFR1 (8p11-13). These rearrangements lead to constitutive activation of downstream signaling pathways that drive eosinophilic proliferation and tissue infiltration. Among them, the FIP1L1-PDGFRA fusion is the most well-characterized and frequently encountered in clinical practice, present in approximately 10-20% of patients with myeloid neoplasms associated with eosinophilia [13].

In our patient, testing of both bone marrow aspirate and peripheral blood resulted in negative findings for the genetic aberrations commonly implicated in myeloid variants of hypereosinophilia. These results were crucial in refining the differential diagnosis and allowed for the exclusion of myeloid/lymphoid neoplasms with eosinophilia (M-HES), which is a group defined by specific fusion genes such as PDGFRA, PDGFRB, FGFR1, and PCM1-JAK2. To further improve diagnostic accuracy, next-generation sequencing (NGS) was performed on a bone marrow sample using the Myeloid Solution Panel (Sophia Genetics®), which screens for mutations linked to myeloid malignancies. No pathogenic mutations were detected in KIT proto-oncogene receptor tyrosine kinase, exon 17 (KIT exon 17), FMS-like tyrosine kinase 3 (FLT3), nucleophosmin 1 (NPM1), or Janus kinase 2, valine-to-phenylalanine substitution at codon 617 (JAK2 V617F), making a myeloproliferative or leukemic cause of eosinophilia unlikely. When no molecular rearrangements are identified, and other hematologic conditions associated with eosinophilia have been excluded, the diagnosis typically narrows to I-HES or CEL-NOS. Differentiating between these two conditions is based on hematologic features: CEL-NOS is defined by having more than 2% blasts in the peripheral blood and/or more than 5% in the bone marrow, but fewer than 20% to rule out acute leukemia [14].

Another variant, L-HES, is characterized by the absence of these molecular rearrangements and by the presence of a T lymphocyte clone secreting interleukin-5 (IL-5), which is a potent growth factor for eosinophils. Skin involvement is more typical of the lymphocytic variant, while cardiac issues such as endomyocardial fibrosis are more common in CEL cases with the FIP1L1-PDGFRA fusion [15-17]. In addition, in our patient, flow cytometry of the bone marrow revealed a small lymphocyte population (2%) without evidence of clonal expansion or lymphoid malignancy. In light of these findings, as suggested by other researchers, the most plausible diagnosis for our patient is I-HES [18].

## Conclusions

Based on the clinical presentation, histopathology, and thorough diagnostic workup, excluding reactive causes and clonal markers, the most likely diagnosis in our patient was I-HES. I-HES is characterized by persistent eosinophilia (≥1.5×10⁹/L) with organ involvement, in the absence of a known cause. Although a diagnosis of exclusion, early identification is crucial to prevent irreversible organ damage. In this case, despite intensive treatment with corticosteroids, hydroxyurea, and tyrosine kinase inhibitors, the patient's condition worsened, progressing to multi-organ failure. Widespread eosinophilic infiltration, particularly in the lungs and heart, indicates a severe, treatment-resistant form of HES. In conclusion, this case highlights the potentially fatal course of HES, especially when the cardiac and neurological systems are involved, and emphasizes the importance of timely, multidisciplinary management using advanced laboratory and clinical tools to improve outcomes.
